# 

**Published:** 1909-01

**Authors:** 


PUBLISHED MONTHLY. ATLANTA SUBSCRIPTION $1.00

### JOURNAL-RECORD OF MEDICINE

Successor to { Atlanta Medical and Surgical Journal, Established 1855. and Southern Medical Record, Established 1870. } 53d Year

Vol. XI. ATLANTA, GA., JANUARY, 1909. No. 10

